# Treatment of Antibody-Mediated Renal Allograft Rejection: Improving Step by Step

**DOI:** 10.1155/2017/6872046

**Published:** 2017-01-31

**Authors:** Nils Lachmann, Michael Duerr, Constanze Schönemann, Axel Pruß, Klemens Budde, Johannes Waiser

**Affiliations:** ^1^Tissue Typing Laboratory, Charité Universitätsmedizin Berlin, Berlin, Germany; ^2^Department of Nephrology, Charité Universitätsmedizin Berlin, Campus Mitte, Berlin, Germany; ^3^University Tissue Bank, Institute of Transfusion Medicine, Charité Universitätsmedizin Berlin, Berlin, Germany

## Abstract

Throughout the past years we stepwise modified our immunosuppressive treatment regimen for patients with antibody-mediated rejection (ABMR). Here, we describe three consecutive groups treated with different regimens. From 2005 until 2008, we treated all patients with biopsy-proven ABMR with rituximab (500 mg), low-dose (30 g) intravenous immunoglobulins (IVIG), and plasmapheresis (PPH, 6x) (group RLP, *n* = 12). Between 2009 and June 2010, patients received bortezomib (1.3 mg/m^2^, 4x) together with low-dose IVIG and PPH (group BLP, *n* = 11). In July 2010, we increased the IVIG dose and treated all subsequent patients with bortezomib, high-dose IVIG (1.5 g/kg), and PPH (group BHP, *n* = 11). Graft survival at three years after treatment was 73% in group BHP as compared to 45% in group BLP and 25% in group RLP. At six months after treatment median serum creatinine was 2.1 mg/dL, 2.9 mg/dL, and 4.2 mg/dL in groups BHP, BLP, and RLP, respectively (*p* = 0.02). Following treatment, a significant decrease of donor-specific HLA antibody (DSA) mean fluorescence intensity from 8467 ± 6876 to 5221 ± 4711 (*p* = 0.01) was observed in group BHP, but not in the other groups. Our results indicate that graft survival, graft function, and DSA levels could be improved along with stepwise modifications to our treatment regimen, that is, the introduction of bortezomib and high-dose IVIG treatment.

## 1. Introduction

Antibody-mediated rejection (ABMR) is one of the most challenging complications following renal transplantation. Paul Terasaki proposed in his humoral theory of transplantation that the majority of transplants are rejected by the action of antibodies, not cells [1]. In a cross-sectional study, we were able to show that about 30% of patients may have HLA antibodies (HLAab) after transplantation [2]. In more than 30% of HLAab positive patients donor-specific HLAab (DSA) were present. Renal allograft survival at 5.5 years after HLAab testing was significantly lower in patients with detectable DSA as compared to HLAab negative patients (49% versus 83%). In a series of 60 patients Sellarés et al. observed that graft failure was caused by ABMR in the majority of cases [3]. To date, plasmapheresis (PPH) together with the application of intravenous immunoglobulins (IVIG) has been the mainstay of ABMR treatment [4, 5]. Over the last years monoclonal antibodies directed against B cells (rituximab) as well as inhibitors of the proteasome (bortezomib) have expanded our therapeutic repertoire [6]. In a previous retrospective analysis, we observed a trend towards an improved graft survival in patients treated with a combination of bortezomib (1.3 mg/m^2^, 4x), low-dose IVIG (30 g), and PPH (6x) as compared to patients treated with the same regimen but a fixed dose of rituximab (500 mg) instead of bortezomib [7]. However, even the bortezomib-based regimen was not sufficient to treat all episodes of ABMR effectively.

IVIG preparations containing the pooled serum IgG fractions from thousands of donors have been used for treatment of various autoimmune diseases for more than 30 years. Usually, low doses of IVIG (0.1–0.2 g/kg) are used to substitute immunoglobulins (“replacement”) in patients with inherited hypogammaglobulinaemia or following removal of immunoglobulins by PPH. Pursuing immunomodulation higher doses (“therapeutic”) are necessary (1-2 g/kg). Mechanistically, the effects of IVIG on the immune system can be differentiated into effects mediated by the dimeric antigen-binding [F(ab′)_2_] fragment and the Fc fragment [8]. F(ab′)_2_-dependent mechanisms include neutralization of pathologic antibodies (anti-idiotypic) and cytokines, depletion of neutrophils and eosinophils, scavenging of anaphylatoxins such as C3a and C5a, and blockade of cellular receptors. More recent research has shown that much of the immunosuppressive effect of IVIG is mediated via the Fc fragment [8]. These effects include upregulation of the inhibitory Fc*γ* receptor Fc*γ*RIIB, downregulation of activating Fc*γ* receptors, reduction of antibody half-life by competition of IVIGs with pathological antibodies for binding to the neonatal Fc receptor which recycles IgG, and expansion of regulatory T-cells. Interestingly, Fc fragment glycosylation including terminal sialic acid residues seems to be crucial for the effectiveness of IVIG [9]. The fact that this important structure is present only in a minority of the total serum IgG pool [9] explains why high doses of IVIG are necessary to achieve therapeutic efficacy.

Based on the abovementioned evidence on the dosage and the underlying mechanism of action of IVIG, we increased the applied IVIG dose from a low-dose (30 g fixed dose) to a high-dose regimen (1.5 g/kg) in July 2010, in order to further improve the efficacy of our bortezomib-based treatment protocol. Here, we report on the long-term efficacy and safety of treatment with bortezomib, high-dose IVIG, and PPH (group BHP). The obtained results are compared with two preceding groups of patients treated either with rituximab, low-dose IVIG, and PPH (group RLP) or with bortezomib, low-dose IVIG, and PPH (group BLP).

## 2. Patients and Methods

Between January 2005 and November 2008 nine consecutive patients with biopsy-proven ABMR were treated with a fixed dose of rituximab (500 mg i.v.), six sessions of PPH (2.5 L/session, 4% albumin), and low-dose (30 g) polyvalent human IVIG (KIOVIG®) after the last PPH (group RLP). Since 2009, all of our patients with a diagnosis of ABMR have received bortezomib-based treatment. However, three patients received rituximab between 2010 and 2013 because of a diagnosis of preexisting polyneuropathy, which is a well-known side effect and consequently a contraindication for bortezomib treatment. Therefore, group RLP finally comprised 12 patients. Between January 2009 and June 2010 eleven consecutive patients received bortezomib (1.3 mg/m^2^ i.v., days 1, 4, 8, and 11), PPH (6x), and low-dose (30 g) IVIG (group BLP). In July 2010 we increased the IVIG dose and treated 11 consecutive patients with bortezomib, PPH (6x), and high-dose (1.5 g/kg) IVIG (group BHP). In order to avoid removal of rituximab and/or IVIG by means of PPH, rituximab was given one day after the last PPH. IVIG treatment was started two days after rituximab, in order to reduce potential adverse interactions between IVIG and rituximab. In addition, we monitored CD19^+^ peripheral B cells following the administration of rituximab. CD19^+^ B cells were either not measurable or slightly above the detection limit following rituximab treatment [0.01/nL (0.00–0.01); normal range 0.1–0.4/nL]. Patients of all groups additionally received a three-day pulse of methylprednisolone (500 mg/day i.v.). After discharge all patients were regularly monitored in our outpatient clinic.

Renal transplantation was performed at the Charité Hospital based on a negative complement-dependent cytotoxicity crossmatch (CDC-XM) with and without dithiothreitol using T- and B-lymphocytes with current and historical serum. In addition, graft allocation was based on a negative virtual crossmatch by considering current and historical unacceptable antigens as defined by Luminex® based single antigen bead assays. Based on this procedure, none of the patients underwent any kind of desensitization before transplantation. Consequently, only patients with de novo DSA were included.

Altogether, 76% (26/34) of all patients received induction therapy with either basiliximab (*n* = 22) or daclizumab (*n* = 4). The remaining eight patients were transplanted before the introduction of basiliximab or daclizumab and received no induction therapy. The distribution of patients, who received no induction therapy, was not significantly different between groups (group RLP *n* = 4/12, group BLP *n* = 3/11, and group BHP *n* = 1/11, *p* = 0.37).

Renal biopsies were taken on indication only. All patients presented with clinically relevant allograft dysfunction after transplant manifesting as an otherwise unexplained increase of serum creatinine (≥0.3 mg/dL), proteinuria (≥1 g/d), or primary nonfunction in the early phase after transplantation. The diagnosis of ABMR was based on the presence of circulating DSA and significant allograft pathology according to the definitions of the Banff classification [10]. C4d staining was done by indirect immunofluorescence on paraffin sections using a polyclonal rabbit anti-human C4d IgG antibody (Biomedica, Vienna, Austria). Only patients who gave their written informed consent were considered eligible for enrollment. All patients were treated in accordance with the Declaration of Helsinki.

Serum samples before and after treatment were screened for HLA antibodies (HLAab) by the Luminex bead-based assay LABScreen® Mixed (One Lambda, Canoga Park, CA, USA). In addition, HLAab specificities were determined by LABScreen Single Antigen beads assay (One Lambda). As an indicator for the antibody level, the normalized MFI was used. HLAab were considered positive when exceeding an MFI value of 500. The DSA showing the highest MFI at the time of ABMR diagnosis (DSAmax) and the MFI sum of all DSA (DSAsum) were tracked to indicate the effectiveness of treatment.

End of follow-up was June 30th, 2016. Renal allograft survival was defined as the interval between diagnosis of ABMR and return to maintenance dialysis treatment or end of follow-up. The estimated glomerular filtration rate (eGFR) was calculated according to the chronic kidney disease epidemiology collaboration (CKD-EPI) formula [11]. All adverse events, abnormal laboratory values, and hospitalizations were tracked from our web-based electronic patient record system “TBase” [12]. Adverse events occurring during the first year after treatment were documented and graded according to the Common Terminology Criteria for Adverse Events (CTCAE) version 3.0 [13]. Normally distributed variables are summarized as mean ± standard deviation. Not normally distributed variables are summarized as median and interquartile ranges. Comparison between groups was carried out using Fisher's exact test for categorical variables and Kruskal-Wallis test with Dunn-Bonferroni post hoc test for continuous variables. Wilcoxon signed-rank test was used for pairwise comparison between different time points. Graft survival was analyzed according to Kaplan-Meier with a log-rank test. A multivariate Cox proportional hazard model was used to identify covariates which independently contribute to allograft loss following ABMR treatment. A probability of less than 0.05 was considered as statistically significant. Statistical analysis was carried out using IBM SPSS Statistics for Windows, Version 22.0 (IBM Corp., Armonk, NY, USA), and STATA 11 IC software (StataCorp., College Station, TX, USA).

## 3. Results

Relevant patient characteristics are shown in Table 1. The interval between transplantation and diagnosis was not different between groups. In all groups there were patients with “early” ABMR occurring during the first year after transplantation and patients with “late” ABMR. Scoring of renal allograft tissue according to the Banff classification revealed no differences between groups except for the transplant glomerulopathy (cg) score, which was higher in group BLP as compared to group BHP (2.2 ± 1.3 versus 0.8 ± 1.3, *p* = 0.049). The applied IVIG dose for group BHP on average was four times as high as that of both other groups [120 g (range: 80–150 g) versus 30 g, *p* < 0.001]. Following diagnosis, all patients received triple drug maintenance immunosuppression comprising a calcineurin inhibitor or mammalian target of rapamycin inhibitor in combination with steroids and mycophenolic acid. In group RLP more patients received tacrolimus as compared to group BLP (100% versus 55%, *p* = 0.01). Median observation time following diagnosis was 101, 88, and 64 months for groups RLP, BLP, and BHP, respectively. Due to the sequential nature of our treatment protocol modifications follow-up was shortest for the BHP group.

**Figure 1:**
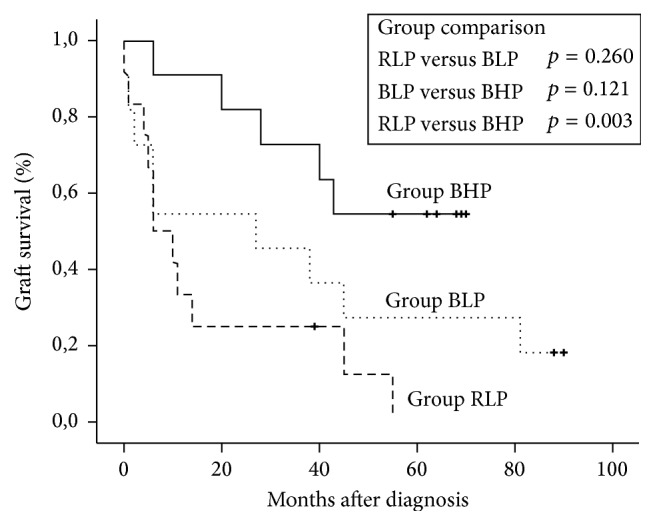
Graft survival according to Kaplan-Meier. Differences between groups were calculated by log-rank test. Note: none of the patients died with a functioning allograft. Group RLP, rituximab + low-dose IVIG + plasmapheresis; group BLP, bortezomib + low-dose IVIG + plasmapheresis; group BHP, bortezomib + high-dose IVIG + plasmapheresis. “+”, end of follow-up.

**Figure 2:**
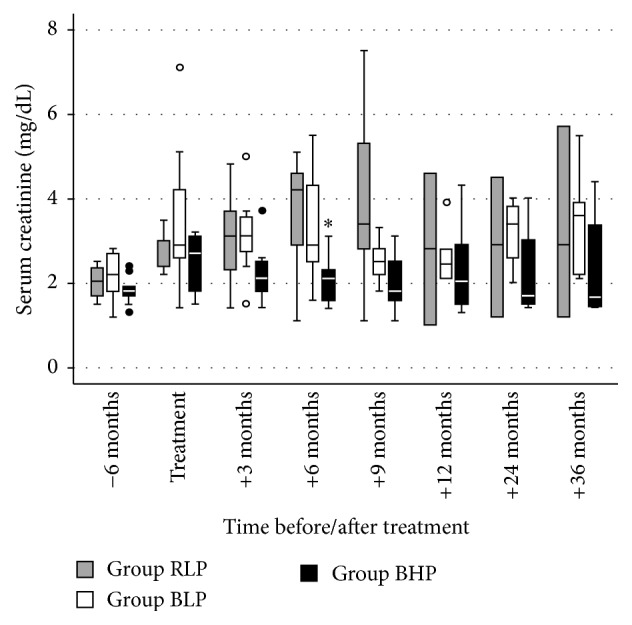
Serum creatinine before, during, and after treatment of antibody-mediated rejection of all patients with a functioning graft at each time point. Differences between groups were calculated by Kruskal-Wallis test with Dunn-Bonferroni post hoc test. Group RLP, rituximab + low-dose IVIG + plasmapheresis; group BLP, bortezomib + low-dose IVIG + plasmapheresis; group BHP, bortezomib + high-dose IVIG + plasmapheresis. ^*∗*^*p* = 0.02 versus group RLP and group BLP.

**Figure 3:**
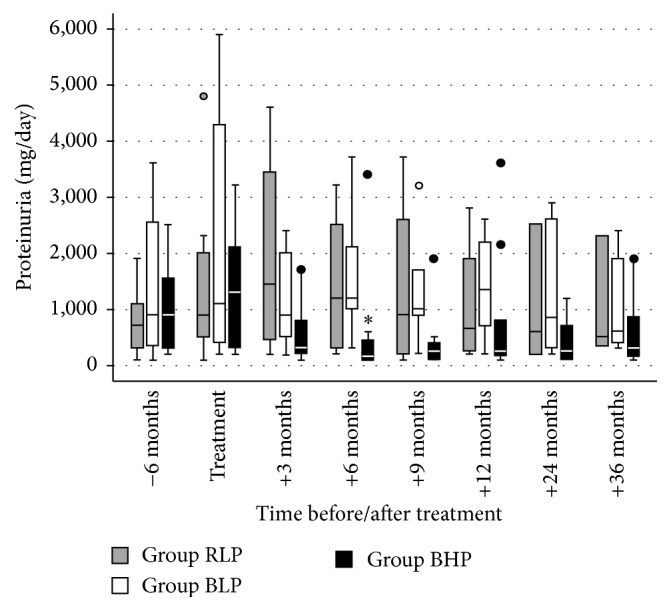
Proteinuria before, during, and after treatment of antibody-mediated rejection of all patients with a functioning graft at each time point. Differences between groups were calculated by Kruskal-Wallis test with Dunn-Bonferroni post hoc test. Group RLP, rituximab + low-dose IVIG + plasmapheresis; group BLP, bortezomib + low-dose IVIG + plasmapheresis; group BHP, bortezomib + high-dose IVIG + plasmapheresis. ^*∗*^*p* = 0.049 versus group RLP and group BLP.

None of the patients died with a functioning graft. Graft survival following diagnosis improved stepwise in the three groups along with the sequential modifications of our treatment protocol, that is, the substitution of rituximab by bortezomib (group RLP → BLP) and the increase of the IVIG dose from a low-dose (30 g fixed dose) to a high-dose (1.5 g/kg) IVIG regimen (group BLP → BHP) (Figure 1). A significant difference in graft survival was observed between group BHP and group RLP (*p* = 0.003). Graft survival at one and three years after treatment was 91% and 73% in group BHP, as compared to 55% and 45% in group BLP and 33% and 25% in group RLP, respectively. At the end of follow-up, graft survival was 55% (6/11) in group BHP, 18% (2/11) in group BLP, and 8% (1/12) in group RLP. At diagnosis, serum creatinine [2.7 mg/dL (1.9–2.9) versus 2.9 mg/dL (2.6–4.1) versus 3.0 mg/dL (2.6–3.0), *p* = 0.23] (Figure 2) and proteinuria [1.3 g/d (0.5–2.1) versus 1.1 g/d (0.45–3.9) versus 0.9 g/d (0.5–1.8), *p* = 0.79] (Figure 3) were not significantly different between groups BHP, BLP, and RLP, respectively. Following treatment, serum creatinine was lower in group BHP as compared to both other groups (Figure 2). A significant difference of group BHP as compared to group BLP and group RLP was observed at six months after treatment [2.1 mg/dL (1.6–2.3) versus 2.9 mg/dL (2.7–3.8) versus 4.2 mg/dL (2.9–4.6), *p* = 0.02] (Figure 2). In accordance, eGFR at six months after diagnosis was higher in group BHP as compared to both other groups [33.0 mL/min/1.73 m^2^ (26.9–41.4) versus 26.0 mL/min/1.73 m^2^ (15.5–32.0) versus 16.3 mL/min/1.73 m^2^ (15.4–19.0), *p* = 0.03]. In addition, proteinuria was significantly lower in group BHP as compared to both other groups at six months after diagnosis [0.15 g/d (0.1–0.4) versus 1.2 g/d (1.0–2.1) versus 1.2 g/d (0.7–2.4), *p* = 0.049] (Figure 3).

The immunological characteristics with respect to HLA mismatches and HLA antibodies are summarized in Table 2. The count of HLA class I and II mismatches was equally distributed between groups. Similarly, HLA antibody panel reactivity (% PRA) at the time of diagnosis did not differ significantly although the HLA class I% PRA among patients of groups BLP and BHP was lower as compared to group RLP (50% and 41% versus 85%, *p* = 0.07). Concerning the level of DSAmax and DSAsum before treatment as indicated by MFI, there was no significant difference between groups. Interestingly, following treatment, DSAmax and DSAsum could be decreased significantly as compared to the pretreatment status in group BHP, but not in the other two groups. The BHP treatment scheme was statistically more efficient in the decrease of DSAmax MFI levels than the RLP scheme [9/11 (82%) versus 4/12 (33%), *p* = 0.04]. In all three groups, there were a few patients with a posttreatment DSAmax level below 500 MFI.

The observed side effects during the first year after treatment are shown in Table 3. The most frequent side effects were haemoglobin reduction (94%), thrombocytopenia (76%), and leukopenia (59%). We observed two cases of grade IV leukopenia in the RLP group and four cases of grade III leukopenia, two of which in the RLP group and two in the BLP group. In the BLP group there were two cases of grade III thrombocytopenia in patients with preexisting thrombocytopenia. All episodes of leukopenia and thrombocytopenia were spontaneously reversible. During episodes of thrombocytopenia no bleeding events were evident. A mild to moderate (grade I-II) increase of transaminase levels occurred in 41% of all patients. Altogether 35 episodes of infection were observed in 16/34 (47%) patients. No difference between groups was found regarding the frequency of infections, neither in general nor with respect to specific infections. Gastrointestinal side effects were observed more frequently in patients, who received bortezomib. In the BHP group significantly more patients suffered from diarrhea as compared to the RLP group (64% versus 8%, *p* = 0.009). Reversible peripheral sensory neuropathy (grade I-II) occurred in two patients of the BHP group. One patient in each group experienced a mild allergic reaction during the IVIG infusion. These events were successfully treated with antihistamines and prednisolone. The number of hospitalizations as well as the number of hospitalized patients was not significantly different between groups. In two patients of group BHP a nonmelanoma skin cancer was diagnosed and successfully treated by local excision.

To correct for any covariate with a potential impact on allograft outcome following ABMR treatment, we performed a Cox proportional hazard analysis with clinical, immunological, and therapeutic covariates as summarized in Table 4. Based on the univariate analysis we identified an impaired allograft function (i.e., eGFR < 30 mL/min/1.73 m^2^) at the time of ABMR diagnosis as well as treatment with rituximab, bortezomib, and high-dose IVIG as the only statistically significant predictors for allograft survival following ABMR treatment. Impaired graft function at diagnosis and ABMR treatment by rituximab were identified as risk factors for subsequent allograft survival. On the contrary, bortezomib and high-dose IVIG revealed a beneficial effect. Subsequent multivariate analysis was performed using impaired graft function at diagnosis and the treatment options rituximab, bortezomib, and IVIG dose as combined covariates. Taken together, impaired allograft function contributed significantly as a risk factor to the regression model (HR = 3.26, 95% CI: 1.29–8.24, *p* = 0.01) and bortezomib plus high-dose IVIG treatment revealed the strongest beneficial effect on allograft survival following ABMR treatment (HR = 0.21, 95% CI: 0.07–0.62, *p* = 0.005) as compared to rituximab plus low-dose IVIG.

## 4. Discussion

The existing literature on the treatment of ABMR is characterized by a marked heterogeneity concerning the definition of ABMR as well as the applied treatment protocols [5]. Notably, a considerable number of the available studies was performed before the introduction of a pathology-based definition for ABMR in 2003 [14]. At that time, various and nowadays outdated criteria to diagnose ABMR were oftentimes used. In addition, new therapeutics more specifically targeting B cell-mediated immune response have become available. The efficacy of these substances as well as their combination with established treatment protocols has not yet been studied sufficiently. A recent randomized study could not demonstrate a benefit of rituximab compared to placebo [15]. Therefore, current evidence for the treatment of ABMR is anything else but satisfactory.

To date, IVIG preparations are widely used for treatment of ABMR [4]. Yet, it is not known, whether high-dose IVIG treatment is advisable in combination with modern antihumoral therapeutics such as bortezomib. Here, we addressed this important question by comparing the efficacy and safety of high-dose IVIG treatment in combination with bortezomib and PPH in 11 consecutive patients with biopsy-proven ABMR (group BHP) with a group of 12 patients treated with low-dose IVIG together with a fixed dose of rituximab and PPH (group RLP) and with a group of 11 patients treated with low-dose IVIG together bortezomib and PPH (group BLP). Group RLP and group BLP have already been partially described in our previous study [7]. Extending the results of this study group RLP now comprises three additional patients, that is, altogether 12 patients, and the median observation time of the present study has been markedly prolonged as compared to our previous study (group RLP: 101 versus 18 months, group BLP: 88 versus 18 months). Notably, the median observation time exceeded five years in all groups. The three groups presented here were comparable regarding the underlying patient characteristics, renal function, and renal pathology at diagnosis except for the transplant glomerulopathy (cg) score, which was significantly higher in group BLP as compared to group BHP.

Our results indicate that treatment with bortezomib in combination with high-dose IVIG and PPH (group BHP) is statistically superior as compared to treatment with rituximab, low-dose IVIG, and PPH (group RLP). In addition, high-dose IVIG with bortezomib and PPH seemed to be advantageous as compared to low-dose IVIG with an identical regimen comprising bortezomib and PPH. Both the change from rituximab-based treatment to bortezomib-based treatment and the increase of the IVIG dose from a low-dose to a high-dose regimen resulted in a stepwise improvement of graft survival and graft function. The observed reduction of DSA following treatment in the BHP group but to a lower extent in both other groups reflects the clinical course and is in concordance with the literature. Terasaki and colleagues demonstrated that a decrease in HLA antibody levels can be used as a surrogate marker for the efficacy of an antirejection treatment and directly correlates with superior allograft survival [16]. Therefore, our results confirm and extend our previous observations [7] inasmuch as bortezomib-based treatment proved to be superior to rituximab-based treatment and that the addition of high-dose IVIG further increased graft survival. Our findings are in line with the results from the randomized RITUX-ERAH trial [15], which demonstrated no statistically significant effects of rituximab for the treatment of ABMR. Our results also suggest that high-dose IVIG seems to be advisable in the presence of modern antihumoral therapeutics such as bortezomib. This conclusion is supported by experimental data showing that only a small proportion of IgG in the available IVIG preparations is responsible for the immunosuppressive effect [8, 9]. Therefore, the amount of applied IVIG seems to be crucial for the success of treatment.

Theoretically, it would have been interesting to investigate a group of patients treated with rituximab, PPH, and high-dose IVIG, in order to further elucidate the efficacy of rituximab and high-dose IVIG treatment. However, in our previous study [7] we observed a clear trend towards an improved graft survival in patients, who received a bortezomib-based treatment over a rituximab-based treatment, so that we deliberately decided to treat all upcoming patients with a bortezomib-based protocol and refrained from further trials on rituximab-based treatment in order not to expose patients to a (in our opinion) potentially higher risk of graft loss.

First reports on the effects of IVIG in the context of renal transplantation have been published 20–30 years ago. In 1984, Steiner et al. investigated the impact of prophylactic IVIG administration on the rate of infection in a prospective randomized trial [17]. Because the proportion of patients discharged with a functioning graft was lower in the treatment group, the authors concluded that prophylactic administration of IVIG is “not only useless but probably dangerous.” About one decade later, Peraldi et al. showed that early high-dose IVIG treatment (2 g/kg) following renal retransplantation improved 5-year graft survival [18]. One of the first reports demonstrating that high-dose IVIG (2 g/kg) may be effective in the treatment of ABMR following kidney transplantation was published by Jordan et al. in 1998 [19]. In 2001, Casadei and colleagues observed that high-dose IVIG treatment (3.5 g/kg) was equally effective in the treatment of steroid-resistant rejection as OKT3 [20]. At the same time, Luke and colleagues also found that high-dose IVIG (2 g/kg) was effective to reverse steroid- or antilymphocyte antibody-resistant rejection [21]. Importantly, a consistent definition of ABMR based on pathological findings and the presence of circulating DSA was not introduced into the Banff classification until 2003 [14]. Consequently, studies performed before 2003 must be regarded with caution. In the more recent era, Lefaucheur et al. reported that treatment of ABMR with high-dose IVIG alone (2 g/kg ×4) was inferior to a combination of high-dose IVIG, PPH, and rituximab, indicating that IVIG alone, even if applied in very high doses, is not sufficient to treat ABMR [22]. In 2014, Cooper et al. showed that high-dose IVIG alone (5 g/kg) caused a modest DSA reduction, especially concerning class I DSA in patients with previous acute ABMR [23].

The effectiveness of IVIG therapy on circulating HLAab has also been investigated in various studies on pretransplant desensitization. In 1993, Glotz et al. described that pretransplant administration of high-dose IVIG (1.6 g/kg) suppresses HLAab formation [24]. More than ten years later, Jordan et al. showed that high-dose IVIG treatment (2 g/kg ×4) reduced PRA levels [25]. In 2006, Stegall et al. observed that high-dose IVIG treatment (2 g/kg) decreased DSA titers [26]. Vo and colleagues reported in 2008 that treatment with high-dose IVIG (2 g/kg ×2) and rituximab (375 mg/m^2^ ×2) reduced PRA levels from 77% to 44% [27]. Recently, the same group compared desensitization with high-dose IVIG (2 g/kg ×2) plus rituximab (1 g) to high-dose IVIG (2 g/kg ×2) plus placebo in a randomized clinical study [28]. Patients received an additional dose of IVIG at transplantation plus an additional dose of rituximab or placebo at six months after transplant. Following transplantation of 13 patients, the study was halted and unblinded because of three episodes of ABMR. All episodes had occurred in the placebo group (3/7) indicating that high-dose IVIG alone did not sufficiently prevent ABMR. Notably, 1/6 patients in the rituximab group died at 12 months after transplantation from a* Nocardia* brain abscess.

In group BLP, two patients received maintenance immunosuppression including everolimus following diagnosis. One of these patients returned to maintenance hemodialysis at 6 months after diagnosis; in the other patient serum creatinine at 88 months after diagnosis is stable at 2.4 mg/dL. In this patient we did not reinitiate calcineurin-inhibitor treatment because of a history of cyclosporine A-induced hemolytic uremic syndrome soon after transplantation. Notably, we were not yet aware of the fact that everolimus-based immunosuppression is associated with an increased risk for the development of DSA and ABMR in 2009 [29]. In addition, a higher cg score was observed in patients of group BLP as compared to group BHP. This might be important, as Billing et al. showed that the degree of transplant glomerulopathy is associated with the response to treatment [30]. Therefore, we included maintenance immunosuppression and cg scores at the time of ABMR diagnosis as covariates in our Cox proportional hazard analysis but could not find a statistically significant impact on allograft outcome.

Taken together, our results demonstrate that graft survival of patients with a diagnosis of ABMR following renal transplantation improved during the past years along with the introduction of bortezomib and high-dose IVIG treatment resulting in a 5-year graft survival of about 50% following diagnosis. In conclusion, the use of high-dose IVIG in combination with a bortezomib-based treatment regimen seems to be useful, especially when compared to a historical group of patients treated with low-dose IVIG, PPH, and rituximab. The degree of DSA reduction in group BHP supports this conclusion and may be used as biomarker for the efficacy of treatment. In our view, stepwise controlled modifications of the established treatment protocols are helpful to gradually improve the prognosis following a diagnosis of ABMR. The main limitation of our study is the fact that it is a retrospective study with a limited number of patients comprising episodes of early and late ABMR and comparing cohorts treated at different time periods that differ in some important variables. Therefore, the results must be interpreted with caution, and further studies are necessary to confirm our findings. In future, IVIG preparations containing a higher proportion of Fc fragments with terminal sialic acid residues may be even more effective. Until such preparations will be available, the amount of applied IVIG seems to be crucial for the success of treatment.

## Figures and Tables

**Table 1:** Patient characteristics.

	Group RLP (*n* = 12)	Group BLP (*n* = 11)	Group BHP (*n* = 11)	*p*
First/repeat transplantation	8/4	7/4	11/0	n.s.
Donor age	52.4 ± 13.8	42.3 ± 14.9	52.1 ± 15.2	n.s.
Living/deceased donor	5/7	5/6	7/4	n.s.
Interval between transplantation and diagnosis (months)	34.6 ± 56.9	58.1 ± 51.4	46.1 ± 44.7	n.s.
Early/late antibody-mediated rejection	6/6	2/9	2/9	n.s.
Pathology scoring				
Glomerulitis (g)	0.8 ± 1.1	0.9 ± 1.0	1.6 ± 1.2	n.s.
Peritubular capillaritis (ptc)	1.1 ± 1.1	0.8 ± 1.1	1.5 ± 1.0	n.s.
Intimal arteritis (v)	0.7 ± 0.9	0.3 ± 0.6	0.5 ± 0.7	n.s.
Transplant glomerulopathy (cg)	1.1 ± 1.2	2.2 ± 1.3	0.8 ± 1.3	0.049 (BLP versus BHP)
C4d (immunohistochemistry)	1.4 ± 1.3	2.0 ± 1.1	1.3 ± 1.3	n.s.
IVIG dose (g)	30	30	120 (80–150)	<0.001 (BHP versus RLP and BHP versus BLP)
Maintenance immunosuppression after diagnosis				
Steroids	12	11	11	n.s.
Cyclosporine A	0	3	1	n.s.
Tacrolimus	12	6	10	0.01 (RLP versus BLP)
Everolimus	0	2	0	n.s.
Mycophenolic acid	12	11	11	n.s.
Median observation time after treatment (months)	101 (39–137)	88 (73–90)	64 (55–71)	<0.001 (BHP versus RLP); 0.015 (BHP versus BLP)

Group RLP, rituximab + low-dose IVIG + plasmapheresis; group BLP, bortezomib + low-dose IVIG + plasmapheresis; group BHP, bortezomib + high-dose IVIG + plasmapheresis. IVIG, intravenous immunoglobulin. Comparison between groups was carried out using Fisher's exact test for categorical variables and Kruskal-Wallis test with Dunn-Bonferroni post hoc test for continuous variables.

**Table 2:** HLA mismatches and HLA antibodies.

	Group RLP (*n* = 12)	Group BLP (*n* = 11)	Group BHP (*n* = 11)	*p*
Number of HLA mismatches per patient (mean ± SD)				
Class I (A, B)	2.2 ± 1.2	2.0 ± 1.3	2.7 ± 0.9	n.s.
Class II (DR, DQ)	1.7 ± 0.9	2.5 ± 1.1	2.0 ± 1.2	n.s.
HLA antibody panel reactivity (% PRA) at diagnosis (mean ± SD)				
Class I	85 ± 14	50 ± 30	41 ± 20	n.s.
Class II	66 ± 20	63 ± 19	63 ± 28	n.s.
DSA_max_ MFI (mean ± SD)				
At diagnosis	8539 ± 5478	9747 ± 8363	8467 ± 6876	n.s.
After treatment	8196 ± 5425	7891 ± 6475	5221 ± 4711^a^	n.s.
DSA_sum_ MFI (mean ± SD)				
At diagnosis	11,235 ± 10,116	12,937 ± 11,196	10,657 ± 8973	n.s.
After treatment	12,900 ± 11,018	12,653 ± 12,744	5587 ± 4509^b^	n.s.
Patients with a decrease of [% (count)]				
DSAmax	33% (4)	72% (8)	82% (9)	0.036 (group BHP versus RLP)
DSAmax < 500 MFI	8% (1)	9% (1)	18% (2)	n.s.
DSAsum	33% (4)	72% (8)	72% (8)	n.s.

Group RLP, rituximab + low-dose IVIG + plasmapheresis; group BLP, bortezomib + low-dose IVIG + plasmapheresis; group BHP, bortezomib + high-dose IVIG + plasmapheresis. DSA_max_, donor-specific HLA antibody showing the highest mean fluorescence intensity (MFI) at time of diagnosis. n.s., not significant. ^a^*p* = 0.01 in comparison to before treatment. ^b^*p* = 0.04 in comparison to before treatment.

**Table 3:** Main adverse events during the first year after treatment.

	Group RLP (*n* = 12)	Group BLP (*n* = 11)	Group BHP (*n* = 11)	*p*
Haemoglobin reduction: baseline – nadir (mg/dL)	2.1 ± 1.5	2.3 ± 1.8	2.8 ± 1.3	n.s.
Thrombocytopenia (patients)	9	10	7	n.s.
Leukopenia (patients)	8	6	6	n.s.
Increase of serum transaminase levels (patients)	6	5	3	n.s.
Infections (events/patients)	14/7	12/7	9/2	n.s./n.s.
Urinary tract infection	7/4	2/1	7/1	n.s./n.s.
Otitis media	2/2	0/0	0/0	n.s./n.s.
Tonsillitis	0/0	1/1	1/1	n.s./n.s.
Pneumonia	1/1	0/0	0/0	n.s./n.s.
Enterocolitis	4/4	4/4	0/0	n.s./n.s.
Central venous catheter infection	0/0	2/2	0/0	n.s./n.s.
CMV reactivation	0/0	2/2	1/1	n.s./n.s.
Fever of unknown origin	0/0	1/1	0/0	n.s./n.s.
Nausea (patients)	0	0	2	n.s.
Vomiting (patients)	0	0	2	n.s.
Diarrhea (patients)	1	2	7	0.009 (RLP versus BHP)
Peripheral sensory neuropathy (patients)	0	0	2	n.s.
Allergic reaction to IVIG (patients)	1	1	1	n.s.
Hospitalizations (events/patients)	16/8	9/7	10/5	n.s./n.s.
Nonmelanoma skin cancer (patients)	0	0	2	n.s.

Adverse events during the first year after treatment are shown except for malignancies, where the whole follow-up period was considered. *Note*. Some patients suffered from more than one gastrointestinal adverse event (nausea, vomiting, or diarrhea) simultaneously. Group RLP, rituximab + low-dose IVIG + plasmapheresis; group BLP, bortezomib + low-dose IVIG + plasmapheresis; group BHP, bortezomib + high-dose IVIG + plasmapheresis.

**Table 4:** Univariate and multivariate cox regression analysis of clinical, immunological, and therapeutic covariates to predict allograft loss following ABMR treatment. Covariates were only considered for the multivariate cox regression model if statistically significant in the univariate analysis.

	HR	95% CI	*p*
Univariate			
Retransplantation	1.70	0.66–4.37	0.27
Induction therapy by IL-2R antibody	0.82	0.31–2.13	0.68
Early ABMR	0.97	0.38–2.47	0.95
eGFR < 30 mL/min/1.73 m^2^ at ABMR diagnosis	2.67	1.07–6.63	**0.03**
Chronic glomerulopathy (cg) score ≥ 1 at ABMR diagnosis	1.69	0.71–4.01	0.23
DSA class I	0.65	0.28–1.50	0.31
DSA class II	0.82	0.30–2.24	0.70
DSA class I + II	0.77	0.32–1.88	0.57
DSAmax > 10,000 MFI	1.57	0.69–3.57	0.28
Everolimus-based maintenance immunosuppression	0.52	0.07–3.94	0.52
ABMR treatment by rituximab	2.74	1.16–6.47	**0.02**
ABMR treatment by bortezomib	0.37	0.15–0.86	**0.02**
ABMR treatment by high-dose IVIG	0.34	0.13–0.93	**0.04**
Multivariate			
ABMR treatment by rituximab + low-dose IVIG (RLP)	1.00	n.a.	n.a
ABMR treatment by bortezomib + low-dose IVIG (BLP)	0.58	0.22–1.52	0.27
ABMR treatment by bortezomib + high-dose IVIG (BHP)	0.21	0.07–0.62	**0.005**
eGFR < 30 mL/min/1.73 m^2^ at ABMR diagnosis	3.26	1.29–8.24	**0.01**

DSA, donor-specific HLA antibody(ies), HR, hazard ratio, MFI, mean fluorescence intensity, n.a., not applicable, 95%CI, confidence interval 95%.

## Abbreviations

ABMR: Antibody-mediated rejection
DSA: Donor-specific HLA antibodies
DSAmax: DSA showing the highest MFI at the time of ABMR diagnosis
DSAsum: MFI sum of all DSA exceeding 500 MFI
HLAab: HLA antibody(ies)
IVIG: Intravenous immunoglobulins
MFI: Mean fluorescence intensity
PPH: Plasmapheresis.
